# Copper Binding and Oligomerization Studies of the Metal Resistance Determinant CrdA from *Helicobacter pylori*

**DOI:** 10.3390/molecules27113387

**Published:** 2022-05-24

**Authors:** Ivana Kekez, Mihovil Faletar, Mario Kekez, Laura Cendron, Maya Wright, Giuseppe Zanotti, Dubravka Matković-Čalogović

**Affiliations:** 1 Department of Chemistry, Faculty of Science, University of Zagreb, Horvatovac 102a, 10000 Zagreb, Croatia; mihovil.faletar94@gmail.com (M.F.); mario.kekez@chem.pmf.hr (M.K.); 2 Department of Biology, University of Padua, Via Ugo Bassi 58/B, 35131 Padua, Italy; laura.cendron@unipd.it; 3 Fluidic Analytics Ltd., Unit A Paddocks Business Centre, Cherry Hinton Road, Cambridge CB1 8DH, UK; maya.wright@fluidic.com; 4 Department of Biomedical Sciences, University of Padua, Via Ugo Bassi 58/B, 35131 Padua, Italy; giuseppe.zanotti@unipd.it

**Keywords:** *Helicobacter pylori*, CrdA, copper binding, stability

## Abstract

Within this research, the CrdA protein from *Helicobacter pylori* (*Hp*CrdA), a putative copper-binding protein important for the survival of bacterium, was biophysically characterized in a solution, and its binding affinity toward copper was experimentally determined. Incubation of *Hp*CrdA with Cu(II) ions favors the formation of the monomeric species in the solution. The modeled *Hp*CrdA structure shows a conserved methionine-rich region, a potential binding site for Cu(I), as in the structures of similar copper-binding proteins, CopC and PcoC, from *Pseudomonas syringae* and from *Escherichia coli*, respectively. Within the conserved amino acid motif, *Hp*CrdA contains two additional methionines and two glutamic acid residues (**M**MX**E**MPGMXX**M**X**E**M) in comparison to CopC and PcoC but lacks the canonical Cu(II) binding site (two His) since the sequence has no His residues. The methionine-rich site is in a flexible loop and can adopt different geometries for the two copper oxidation states. It could bind copper in both oxidation states (I and II), but with different binding affinities, micromolar was found for Cu(II), and less than nanomolar is proposed for Cu(I). Considering that CrdA is a periplasmic protein involved in chaperoning copper export and delivery in the *H. pylori* cell and that the affinity of the interaction corresponds to a middle or strong metal–protein interaction depending on the copper oxidation state, we conclude that the interaction also occurs in vivo and is physiologically relevant for *H. pylori*.

## 1. Introduction

Regulation of metal ion homeostasis is of critical importance to all living organisms. Adaptation of *Helicobacter pylori* to the conditions in the gastric mucosa involves acquisition mechanisms that can overcome a temporary lack of essential metal ions. Copper ion is an important cofactor in the regulation of metal homeostasis in *H. pylori* and plays a significant role in electron transport, oxidases, and hydroxylases, and can mediate the formation of oxygen reactive species. Several proteins are involved in the transport of copper ions and in control of the concentration of free copper ions in the cytoplasm below toxic values. Among them are P-type ATPase CopA [1], HP1326 (CrdA), HP1327 (CrdB), HP1328, and HP1329 [2]. Histidine, cysteine, and methionine are the most common residues found in the binding sites of cuproproteins. In plastocyanin, a copper center is coordinated by two histidines, methionine and cysteine, in a distorted tetrahedral geometry [3]. In the case of some cuproproteins, such as umecyanin and cytochrome c oxidase, other amino acid residues, such as glutamic acid, also participate in copper binding with a tetrahedral coordination environment Cu(N^δ^_His_)_2_S^γ^_Cys_O^ε^_Glu_ and *μ*(S^γ^_Cys_)_2_(CuN^δ^_His_S^δ^_Met_)(CuN^δ^_His_O_Glu_), respectively [4,5,6].

CrdA from *Helicobacter pylori* (*Hp*CrdA) is a 13.8 kDa putative copper-resistance determinant, rich in methionines, and is required for keeping the concentration of free copper ions below toxic levels [7]. In contrast to known copper chaperones, the protein sequence of *Hp*CrdA lacks cysteine and histidine residues. Since *Hp*CrdA is a secreted protein, it is predicted that the first 20 amino acids are the signal peptide. Protein sequences of *Ps*CopC and *Ec*PcoC from *Pseudomonas syringae* and *Escherichia coli*, respectively, share with *Hp*CrdA a common MXXMPGM amino acid motif. Crystal structures of the copper-binding protein *Ps*CopC (PDB ID: 2C9Q) [8], and *Mt*CopC from *Methylosinus trichosporium* (PDB ID: 5ICU) [9], revealed that these proteins could contain different binding arrangements: canonical two copper-binding sites, (Cu(II) and Cu(I))–category C_1-1_ CopC), or just a single Cu(II) binding site (Cu(II)–category C_0-1_ CopC), respectively. Similarly, *Mt*CopC, CopC proteins from *Pseudomonas fluorescens* SBW25 (*Pf*) [10] and *Thioalkalivibrio paradoxus* ARh 1 (*Tp*) [11] lack the Cu(I) binding site and bind only Cu(II). Sequence analyses of the C_0-1_ CopC proteins showed that the conserved Met residues necessary for Cu(I) binding are missing [10]. According to this classification, there are also C_0-0_ (copper non-binding) and C_1-0_ (only Cu(I) binding) CopC proteins [9]. Brander et al. [12] showed that *Pf*CopC is a strong Cu(II)-chelator due to partial deprotonation of the N-terminus by Glu27 and enabled by Ala2 and, moreover, that *Pf*CopC can also bind Cu(I) but with much lower affinity than the sub-femtomolar Cu(II) affinity previously reported [13].

PcoE from *Escherichia coli* (*Ec*PcoE) is a protein involved in a rapid copper response via the initial sequestration of copper in the periplasm, while the remaining genes of the Pco system (*pcoABCD*) are being fully induced [14]. Interestingly, despite the fact that the overall sequence similarity with the *Hp*CrdA is low, the sequence of *Ec*PcoE is also rich in Met (12%) residues. It was shown that PcoE could bind multiple metal ions with varying affinities, with the highest affinity being for Cu(I) in the picomolar range. The affinity of *Ec*PcoE toward Cu(I) is lower than that of *Ec*PcoC, a potential protein partner. CusF from *Escherichia coli* (*Ec*CusF) is a periplasmic metallochaperone [15] and is highly specific for the monovalent metal ions (Cu(I) and Ag(I)) [16,17,18]. *Ec*CusF binds Cu(I) via a Met_2_His motif. The crystal structure of *Ec*CusF (PDB ID 2VB2) revealed one copper-binding site. In the crystals grown in excess of Cu(II), the binding site accommodates Cu(II), but this site is proposed to be the binding site for Cu(I) [19]. A similar analysis applies to the crystal structures of *Ps*CopC (PDB IDs: 2C9Q, 2C9P) [8], where Cu(II) is bound in the canonical Cu(I) site.

At present, no structural data for *Hp*CrdA are available, and the only study is on RNA profiling [2]. According to a former study, ORF HP1326 that encodes the *H. pylori* CrdA protein showed a strong transcription activity upon increased copper supplementation, while the Pfr synthesis was repressed. Similar behavior was observed when the neighboring genes, HP1327 (CrdB) and HP1328 (CzcB), were inactivated. Other homologs of known copper regulation activity, such as *Escherichia coli* CueR, *Pseudomonas* CopR/S, and *Ralstonia* CzcRS, are not present in the *H. pylori* genome. Taking all evidence into account, Waidner et al. (2002) [2] proposed a novel type of copper efflux pump called the Czc system that consists of copper resistance determinants, CrdA (HP1326), CrdB (HP1327), CzcB (HP1328), and CzcA (HP1329), which *H. pylori* require for keeping the concentration of free copper ions below toxic levels.

This work is part of our research on *Helicobacter pylori* proteins [20,21]. Our goal was to characterize *Hp*CrdA biophysically and structurally in a solution to consider its biophysical and structural properties and to define its function. The structural characterization was performed by a biophysical and structural analysis utilizing the following methods: thermal stability assay, circular dichroism (CD), and 1D/2D-NMR. To understand if *Hp*CrdA exerts the function of copper transport and whether it contains a canonical two or one copper-binding site, the interaction between copper ions and *Hp*CrdA was investigated by UV–VIS titration and microscale thermophoresis (MST).

## 2. Materials and Methods

### 2.1. Molecular Cloning

The *hp1326* gene was cloned from the purified genomic DNA of the *H. pylori* strain, G27. Cloning without the first 22 amino acids was performed since the *Hp*CrdA sequence analysis, by the *LipoP* bioinformatic tool [22], predicted a cleavage site for the export signal and a lipidation site (aa 22−23). The *Hp*CrdA construct was inserted into the pGEX-6P-1 (GE Healthcare) vector in frame with the N-terminal GST-tag that was previously linearized with restriction endonucleases, *Bam*HI and *Xho*I, and the gene of interest (*hp1326*) was amplified using primers (forward: 5′ GAGAGAGGGATCCATGCAAACCCTAAAAGCCAAC 3′, reverse: 5′ GAGAGAGCTCGAGTTATAAATCCAGGCTTGTTTTTAG 3′) that introduced an N-terminal *Bam*HI recognition site and a C-terminal *Xho*I site to allow directed integration into the vector.

### 2.2. Expression and Purification

*Escherichia coli* BL21(DE3) cells were transformed with pGEX-6P-1-*Hp*CrdA and were grown at 37 °C in 4 L of Luria Bertani (LB) medium supplemented with 50 µg mL^−1^ ampicillin. When the OD_600_ of 0.6 was reached, the protein expression was induced by adding 0.5 mM isopropyl-β-d-1-thiogalactopyranoside (IPTG) at 16 °C. After induction overnight, the cells were harvested by centrifugation, resuspended in a buffer (20 mM Tris-base pH 7.5, 150 mM NaCl, 2 mM phenylmethylsulfonyl fluoride (PMSF)), and disrupted with the One Shot Cell breakage system (Constant System Ltd., Daventry, UK; 1.36 kbar). The lysate of GSTCrdA from *H. pylori* (*Hp*GSTCrdA) was clarified by centrifugation (30 min at 40,000 g), and the soluble fraction was loaded onto a 1 mL GSTrap column *(GE Healthcare*), equilibrated with buffer A (140 mM NaCl, 2.7 mM KCl, 10 mM Na_2_HPO_4,_ 1.8 mM KH_2_PO_4_ pH 7.5). After washing with buffer A, the protein was eluted with buffer E (10 mM reduced gluthatione 50 mM Tris-HCl pH 8.0) and further treated with PreScission protease to cleave the N-terminal GST-tag from the fusion protein. The protein:protease mass ratio was adjusted to 1:200 in a buffer containing 50 mM Tris-base pH 7.5, 50 mM NaCl, 1 mM ethylenediaminetetraacetic acid (EDTA), 1 mM dithiothreitol (DTT), and a 3.8 mM n-octyl-β-d-glucopyranoside detergent (nOG). The sample was incubated at 4 °C overnight. Afterward, *Hp*CrdA was concentrated by ultrafiltration (Vivaspin 15R 10,000 MWCO, Sartorius) and further purified by using the size-exclusion column Superdex 75 10/300 GL (GE Healthcare).

The purity of the samples during all of the purification steps was verified by electrophoresis under denaturing conditions (SDS-PAGE).

### 2.3. Characterization in Solution

The monodispersity of the *Hp*CrdA protein treated with different detergents was evaluated at a concentration of 2 mg mL^−1^ in buffer B (50 mM Tris-base pH 7.5, 50 mM NaCl) and at 25 °C by dynamic light scattering (DLS, Zetasizer Nano ZS, Malvern Instruments Ltd.). Prior to their use, the samples were centrifuged for 10 min at 10,000 rpm, and 40 µL of supernatant was transferred into a quartz ZEN 2112 cuvette.

A secondary structure analysis of the diluted *Hp*CrdA (at a concentration of 0.5 mg mL^−1^ in buffer B) was performed by circular dichroism (CD) using a spectropolarimeter (Jasco Analytical Instruments) in the far UV region (190−260 nm). Afterward, the data were deconvoluted using the software CDNN [23].

2D-NMR was used for the evaluation of the presence of secondary structure elements of the cleaved sample of *Hp*CrdA (at a concentration of 0.1 mM in buffer B supplemented with 3.8 mM nOG). A 15N HSQC spectrum of the *Hp*CrdA protein sample was recorded on an 800MHz VNMRS NMR spectrometer using an HCN cryogenic probehead with the inverse detection at 298K.

The average hydrodynamic radius of *Hp*CrdA (at a concentration of 0.1 mg mL^−1^) was assessed using microfluidic diffusional sizing on a Fluidity One (Fluidic Analytics Ltd., Cambridge, U.K.) in the 1–8 nm flow rate range. Three repeats were taken on 5 μL of *Hp*CrdA in 50 mM Hepes, 150 mM NaCl, 1 mM nOG, and 5% (*v*/*v*) glycerol.

In order to examine the oligomeric state of the *Hp*CrdA protein incubated with EDTA prior to titration (see Section 2.4), as well as after titration with copper(II) ions (no EDTA after the buffer exchange), analytical size-exclusion chromatography (SEC) was carried out by using a Superose 12 10/300 GL (GE Healthcare) column equilibrated with a buffer containing 20 mM Tris-base pH 7.5, 150 mM NaCl, and 3.8 mM nOG. The same size-exclusion protocol was applied for the protein molecular weight standards: vitamin B12 (1.35 kDa), myoglobin (17 kDa), ovalbumin (44 kDa), and γ globulin (158 kDa). The void volume was determined by using Blue Dextran (GE Healthcare).

Thermal stability assays were carried out to investigate the protein stability after the addition of different metal ions while the buffer condition was kept constant. 20 µL of *Hp*CrdA, at a concentration of 1 mg mL^−1^ in buffer B supplemented with 3.8 mM nOG, was mixed with 1 µL of the selected metal ion solution (M^2+^) at a concentration of 100 mM (M^2+^ = Cu, Mg, Ni, Zn) and incubated for 5 min. Afterward, the capillaries were filled with the mixture and ran on the Tycho NT.6 instrument (Nanotemper Technologies). *Apo*-*Hp*CrdA was run as a control.

### 2.4. Ultraviolet-Visible (UV-VIS) Titration

UV–VIS titration was performed to investigate the affinity of the *Hp*CrdA protein toward copper(II) ions. Before performing the titration experiment, *Hp*CrdA was incubated with 10 mM EDTA to remove any possibly bound metal ions, and then the buffer was exchanged for the buffer containing 30 mM Na_2_HPO_4_ pH 7 and 50 mM NaCl. Copper titrations were performed on an Agilent 8453 UV–vis Spectroscopy *System* at room temperature in the wavelength range of 200−800 nm. 1mL of *Hp*CrdA (150 µM) was titrated with CuSO_4_ using a stock solution at a concentration of 4 mM. All of the spectra were blanked against the used buffer. The reaction mixture was incubated for 5 min after every new addition of titrant before measuring the absorbance. The raw data were analyzed in GraphPad Prism software 6 [24] using the nonlinear regression binding-saturation one site-total function.

### 2.5. Microscale Thermophoresis (MST)

MST experiments were carried out with a Monolith.NT115 instrument (MO. Affinity Analysis 2.3 NanoTemper Technologies GmbH, Münich, Germany) at 25 °C. The *Hp*CrdA was labeled according to the manufacturer’s instructions with 647-NT-NHS red fluorescent dye (NanoTemper Technologies). During the labeling procedure, the protein was incubated with EDTA to remove any trace of metal ions and, afterward, the buffer was exchanged for the assay buffer (20 mM Mops, pH 7.5, 150 mM, NaCl, and a 0.05% (*w*/*v*) Tween detergent). A stock solution of copper(II) chloride (4 mM) was serially diluted in the assay buffer up to a volume of 10 μL. Afterward, 10 μL of the fluorescently labeled *Hp*CrdA protein were added, and the mixture was incubated on ice for 10 min to allow the formation of the protein complex, transferred to Premium capillaries, and analyzed for fluorescence response in a microtemperature gradient. The labeled protein was added in an amount to efficiently gain fluorescence response in the range of 200–1500 response units. Instrument parameters were adjusted with 20% LED power and 20 to 40% MST power. Measurements were performed at least three times. The data was analyzed in the GraphPad Prism software 6 [24] for the calculation of the *K*_d_ values using a non-linear regression: one site total.

The concentration of all of the stock copper(II) chloride solutions was determined by inductively coupled plasma optical emission spectroscopy on an inductively coupled plasma optical spectrometer, the Prodigy High Dispersion ICP (Teledyne Leeman Labs., Hudson, NH, USA). The instrument is equipped with 40 MHZ “free-running” radiofrequency generator and echelle grating spectrometer with a large-format programmable array detector (L-PAD). The dual-view torch for observing both the axial and radial position was used. The sample solution uptake rate was adjusted to 0.9 mL min^−1^. The generator power of 1.2 kW and flow rates of argon (coolant 18 L min^−1^ and auxiliary 0.8 L min^−1^) were held constant during the measurements. The copper emission line at 324.754 nm, free from spectral and background interferences, was selected from the image. The precision of the intensity measurements on the chosen analytical emission line was 0.8% RSD; the detection limit (LOD), based on the 3σ criterion, comprised 0.7 µg L^−1^.

## 3. Results and Discussion

### 3.1. Expression Level of HpCrdA

The best condition for expressing the *Hp*GSTCrdA protein was obtained when *E. coli* cells were grown at 16 °C. As can be seen in Supplementary Materials Figure S1, the SEC (Superdex 75 10/300 column) of the cleaved *Hp*GSTCrdA was successfully applied for the separation of the *Hp*CrdA protein from the GST-tag protein. This was possible since the GST protein is present as a dimer in the solution (in which the molecular mass of the monomer was ~26 kDa) while *Hp*CrdA is a monomer (in which the molecular mass of the monomer was ~12.4 kDa).

### 3.2. Size of HpCrdA in Solution

**Figure 1:**
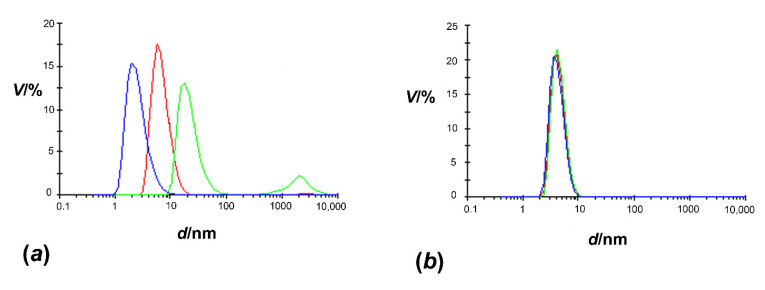
The volume distributions as a function of particle diameter: (**a**) *Hp*CrdA and (**b**) *Hp*CrdA treated with nOG detergent; *c* = 3.8 mM. Each measurement was recorded three times (blue, green, and red curves).

As can be seen in Figure 1, the dispersity of pure *Hp*CrdA and of the sample treated with the nOG detergent (*c* = 3.8 mM) differs significantly. The solution of the *Hp*CrdA protein without detergent was heterogeneous and contained species in different oligomeric states and with a high level of aggregation. The polydispersity index for the *Hp*CrdA sample and *Hp*CrdA plus nOG was 0.941 and 0.335, respectively, implying that only the sample treated with detergent could be used for further experiments. The influence of other detergents, such as LDAO (*c* = 0.4 mM), DDM (*c* = 0.034 mM), and Tween (0.05% (*w*/*v*) on the dispersity of *Hp*CrdA was also tested; however they only slightly affected the dispersity profile of *Hp*CrdA.

**Figure 2:**
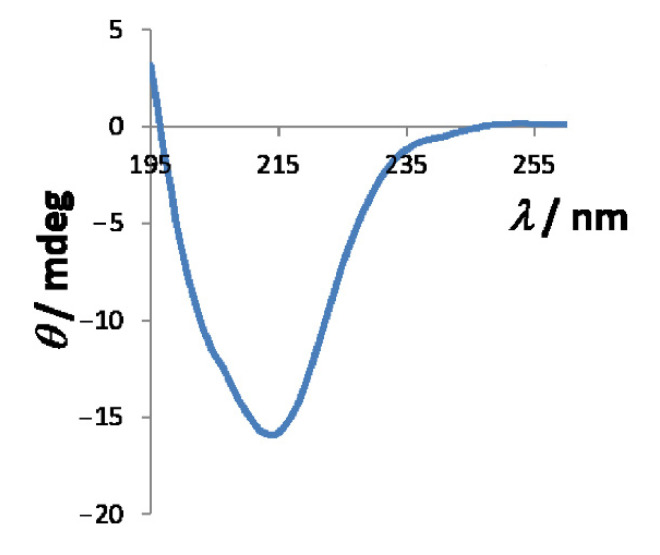
CD spectrum of *Hp*CrdA in the far UV region (195−260 nm) presented as ellipticity in milidegrees.

The proper folding of the cleaved *Hp*CrdA was confirmed by CD and 2D-NMR spectroscopy (Figure 2 and Figure S2). The far-UV circular dichroism spectrum (Figure 2) shows a minimum at 214 nm and a peak of positive ellipticity for the values below 197 nm that are characteristic of a properly folded protein rich in β sheets (Table S1) [25]. The 2D-NMR spectrum also confirms proper protein folding since the signal dispersion in the proton dimension is well distributed. Additionally, the 2D-NMR spectrum shows many signals >8.5 ppm in the proton dimension (Figure S2), which is characteristic of proteins rich in β-strands as confirmed with CD analysis.

The average hydrodynamic radius of *Hp*CrdA was assessed using microfluidic diffusional sizing. This method allowed in-solution sizing using post-separation labelling on a microfluidic chip. The average radius of the *Hp*CrdA obtained was 1.92 +/−0.02 nm for the triplicate measurements (Figure S3), and the estimated molecular weight was ~13.1 kDa, assuming a fully folded globular protein structure.

**Figure 3:**
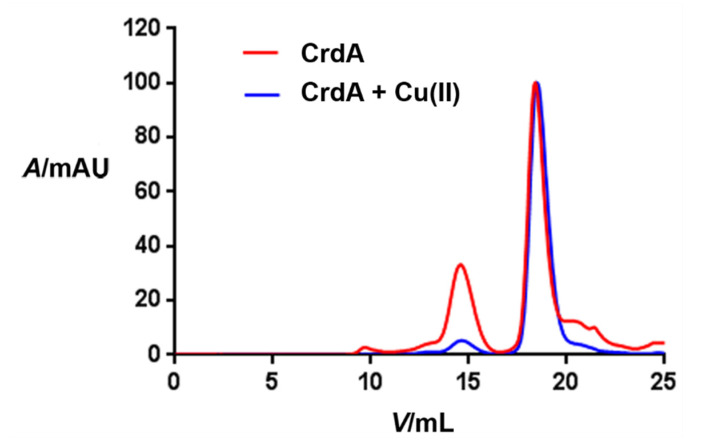
Analytical gel filtration chromatogram of *Hp*CrdA titrated with Cu(II) ions (blue curve; predicted molecular mass of the monomer is ~12.4 kDa), which elutes with a major peak at the volume of 18.5 mL corresponding to the molecular mass of 14.8 kDa. *Hp*CrdA chelated with EDTA (red curve) predominantly elutes as a monomer as well but shows a higher contribution of the dimeric species (these two different species elute at 14.5 mL and 18.5 mL and correspond to the molecular mass of 31 kDa and 14.8 kDa, respectively).

The incubation of *Hp*CrdA supplemented with nOG and with copper(II) ions or EDTA, followed by SEC, was carried out. *Hp*CrdA treated with copper(II) was eluted as a single peak at a volume of 18.5 mL (only a very small peak at 14.5 mL was obtained), while in the case of *Hp*CrdA treated with EDTA two different species were eluted: one at 14.5 mL and the other one at 18.5 mL (Figure 3). The calculated molecular mass from the SEC experiments of the *Hp*CrdA species in the presence of copper ions was about 14.8 kDa, a value in close agreement with the predicted molecular mass of 12.4 kDa and with a molecular mass of 13.1 kDa obtained using microfluidic diffusional sizing. The observed molecular mass indicates that the *Hp*CrdA protein treated with copper(II) is present as a monomeric species in the solution, being consistent with the analytical gel-filtration results obtained for the copper-binding CopC protein from *Pseudomonas syringae* (*Ps*CopC) in the solution [8]. In the case of treatment with a metal chelating agent (EDTA), the *Hp*CrdA protein eluted in two different oligomeric states-monomeric (14.8 kDa) and dimeric (31 kDa), indicates an equilibrium between these two oligomeric species. For *Ec*PcoE, it was found to remain monomeric upon titration of Cu(II) into apo-PcoE in the solution [14]. In the case of PcoC from *Escherichia coli* (*Ec*PcoC), analytical ultracentrifugation experiments showed a self-associating monomer-dimer equilibrium, and upon the addition of copper(I), the equilibrium significantly shifted toward dimerization [26].

### 3.3. Affinity of HpCrdA towards Copper(II) Ions

**Figure 4:**
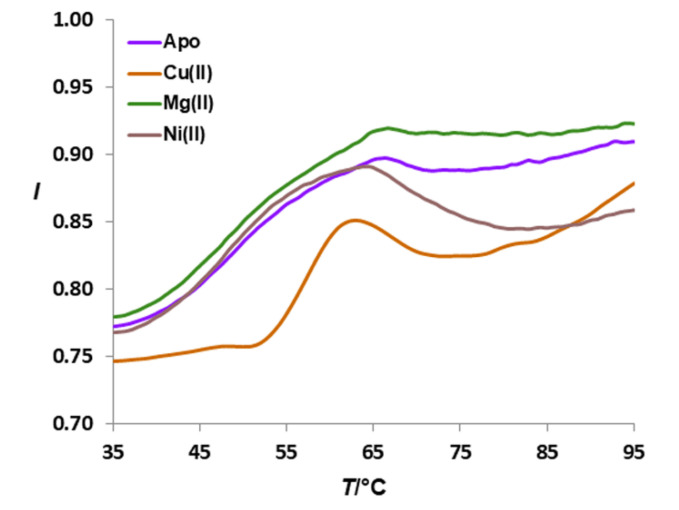
The stability assay of *Hp*CrdA supplemented with different metal ions (Tycho NT.6 instrument). Fluorescence intensities (*I*) were measured at a 350/330 nm ratio.

In order to confirm the affinity of *Hp*CrdA toward the copper(II) ion, we have performed screening of different metal ions using the thermal stability assay. The sample with Zn^2+^ denatured the protein, so it could not be used for thermal stability determination. Thermal stability experiments showed a stability shift of +8.1 °C (Figure 4) only for the sample treated with Cu(II) ions (orange curve), so this metal was chosen for further *Hp*CrdA:metal binding experiments.

**Figure 5:**
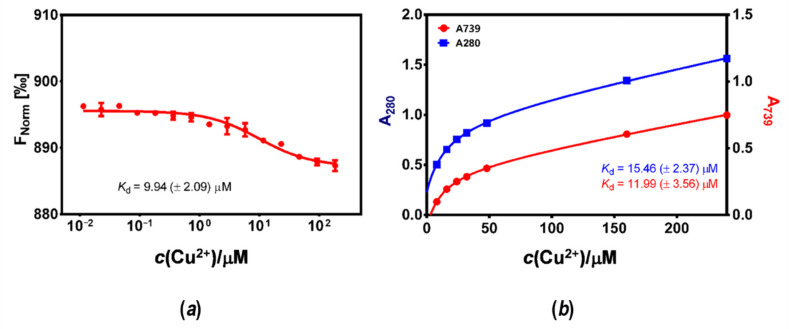
(**a**) Interaction study of the Cu(II):*Hp*CrdA complex by microscale thermophoresis. The signal is in parts per thousand. The raw data were analyzed using the dose-response sigmoidal fit function, and the dissociation constant (*K*_d_) was determined. The data are the mean from two independent experiments (red curve). (**b**) Change in the UV–VIS spectrum upon titration of *Hp*CrdA with an increasing concentration of copper(II) ions. Plot of *A*_280_ and *A*_739_ versus *c*(Cu^2^^+^) are shown in blue and red curves, respectively.

The association of *Hp*CrdA and copper ions was investigated using microscale thermophoresis (MST). The thermophoretic mobility of fluorescently labeled *Hp*CrdA in the presence of increasing concentrations of copper(II) ions was monitored (Figure 5a), and the *K*_d_ value of the interaction was found to be 9.94 (±2.09) μM. To further investigate the copper-binding properties of the *Hp*CrdA protein, titration monitored by UV–VIS spectroscopy was carried out. The addition of copper(II) ions to *apo*-*Hp*CrdA induced an increase in the absorbance of around 280 nm and 740 nm. The absorbance at 280 nm is plausibly due to the ligand Met Cu(II) charge transfer transition [8]. The determined *K*_d_ for the Cu(II):*Hp*CrdA interaction was 15.46 (±2.37) μM and 11.99 (±3.56) μM at *A*_280_ and *A*_739_, respectively (Figure 5b). These *K*_d_ values are similar to the *K*_d_ value obtained from the MST experiment. The crystal structure of the wild-type Cu(I)Cu(II)-*Ps*CopC revealed two separated binding sites with high affinities for Cu(I) (10^−7^ ≥ *K*_d_ ≥ 10^−13^ M) and Cu(II) (*K*_d_ = 10^−13(1)^ M), implying that CopC is a copper scavenging protein in the oxygenated periplasm [8]. The variant forms of His1Phe-*Ps*CopC and His91Phe-*Ps*CopC showed a lower affinity for Cu(II) than the wild-type protein (*K*_d_ > 10^−5^ M for the His1Phe-*Ps*CopC variant). However, in the crystal structure of *Ec*PcoC, no copper was found, even after soaking crystals in a solution of Cu(II) or Cu(I) ions [26]. The crystal structure of *Ec*CusF (PDB ID 2VB2) revealed one copper-binding site. In the crystals grown in excess of Cu(II), the binding site accommodates Cu(II), but this site is proposed to be the binding site for Cu(I) [19]. *Ec*CusF binds Cu(I) in a nanomolar range, as determined by ITC (isothermal titration calorimetry) [16,18]. The binding affinity of Cu(I) for *Ec*PcoE was measured in the picomolar range, and it was shown that *Ec*PcoE could also bind Cu(II) but with a lower affinity [14]. The equilibrium dissociation constants for the Cu(II):*Hp*CrdA complex determined by MST and UV–VIS spectroscopy indicate a middle-affinity metal–protein interaction, which is characteristic of copper trafficking proteins rather than metalloproteins with an enzymatic function [4]. By MST measurements (Figure S4) for the Cu(I):*Hp*CrdA complex, we were not able to detect the interaction in the nanomolar range. There is a possibility that *Hp*CrdA binds Cu(I) in a range lower than nanomolar.

### 3.4. HpCrdA Modeling

**Figure 6:**
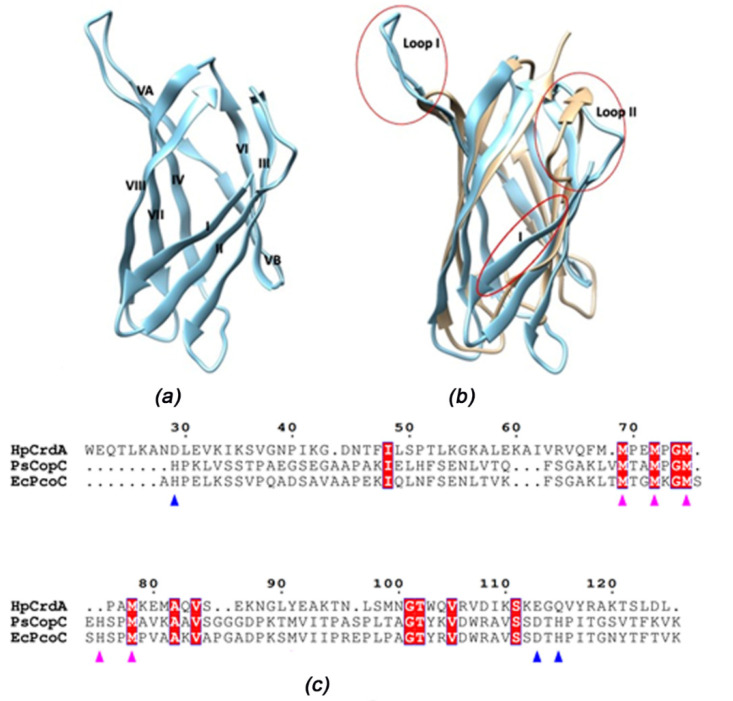
*Hp*CrdA model in comparison with the *Ps*CopC crystal structure. (**a**) Cartoon view of *Hp*CrdA, without residues 1-21 that are predicted to be as an export signal. Strands are numbered from I to VIII. (**b**) Superposition with the *Ps*CopC crystal structure. Regions significantly different in the two models are circled. (**a**,**b**) were prepared using the Chimera molecular graphics tool [30]. (**c**) Structural sequence alignment of *Hp*CrdA (AF-O25884-F1-model-v2), *Ps*CopC (P12376) and, *Ec*PcoC (Q47454). The alignment figure was generated by superimposing the AlphaFold-2 model of *Hp*CrdA to the crystal structures 2C9Q and 1LYQ. The numbering system on the top line is that of the *Hp*CrdA sequence. Residues fully conserved in all three sequences are labelled in white and highlighted in red. Possible ligands for the Cu(II) and Cu(I) ions in two sites according to the *Ps*CopC structure [8] are marked with blue and magenta triangles, respectively. (**c**) was prepared using ESPript [31].

The predicted structure of *Hp*CrdA was downloaded from the AlphaFold 2 server (AF-O25884-F1-model-v2) [27,28]. The model of *Hp*CrdA is a single domain with a Greek key *β−*barrel fold [29] that agrees with the CD data. At variance with that of other members of this family, such as the *Pseudomonas* and *E. coli* structures, it presents an additional *β*-strand at the N-terminus (strand I). In this way, the *Hp*CrdA barrel includes eight strands instead of seven (Figure 6a). Overall, there are several significant differences in *Hp*CrdA compared with the available crystal structures, particularly in the loops connecting strands II and III (op I-corresponding to strands I and II in CopC) and strands IV and V (loop II; Figure 6b). This is clearly reflected by the root mean square deviation (r.m.s.d.s) values between the Cα atoms: the superposition of *Hp*CrdA to *Ps*CopC (PDB ID 2C9Q) and *Ec*PcoC (1LYQ) gives an r.m.s.d. of 2.23 Å and 2.42 Å for the alignment of 71 and 76 residues, respectively. The r.m.s.d between the two *Ps*CopC and *Ec*PcoC crystal structures is 0.99 Å for 100 residues, i.e., the entire structures.

The alignment of the amino acid sequence of *Hp*CrdA with those of *Ps*CopC and *Ec*PcoC based on the superposition of the structures is shown in Figure 6c. The very limited number of fully conserved amino acids reflects significant differences in the 3D structures.

**Figure 7:**
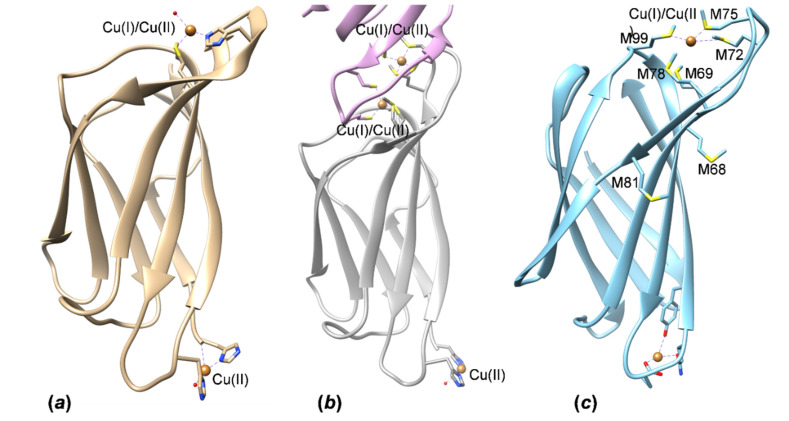
Cartoon view of copper-binding sites in CopC family members. (**a**) The binding sites of Cu(II) and Cu(I) in CopC from *Pseudomonas syringae* at high pH (PDB ID 2C9Q) and (**b**) low pH (monomers A and B in pink and grey color, respectively; PDB ID 2C9P). Cu(I)/Cu(II) is the label for the copper atom, which is Cu(II) in the crystal structures but is proposed to be the binding site for Cu(I). (**c**) Model of *Hp*CrdA with the hypothetical position of the Cu(I) and Cu(II) binding sites. The figure was prepared using Chimera molecular graphics tool [30].

In *Ps*CopC, the regions involved in the binding of Cu(II) and Cu(I) are located on opposite sides of the *β*-barrel (Figure 7a,b). The main binding site for Cu(II) is similar to the three available crystal structures of the bacterial proteins determined in the presence of copper; those of *Pseudomonas syringae* at a high and low pH (pH ~ 7.5, PDB ID 2C9Q; pH ~ 4.6, PDB ID 2C9P) (Figure 7a,b) and *Pseudomonas fluorescens* (PDB ID 6NFQ) (Figure S5). The Cu(II) ion is coordinated by two histidines and the third site is occupied by a water molecule or an aspartate residue in *Ps*CopC and *Pf*CopC, respectively (Figure 7a,b and Figure S5). The corresponding residues in the same area in *Hp*CrdA are Glu113, Gln115, and Tyr117: their chemical nature is different, except for the conserved glutamate, but their side chains are properly oriented and might coordinate a metal ion (Figure 7c). This could be a binding site for Cu(II) in a high concentration, but this site is more suitable for a harder metal ion [32].

More interesting appears the presence of a conserved sequence of the group of methionines in *Hp*CrdA. They are present also in CopC and in the crystal structure of *Ps*CopC at a low and high pH and have been demonstrated to be involved in the binding of Cu(I) despite the fact that, in the crystal structures, the Cu(II) ion is bound at this site. In the high pH structure, the copper ion is coordinated by a histidine, methionine, and a water molecule, whereas in the low pH structure, this binding is performed through the formation of a protein dimer, where two copper ions, one from each monomer, are tetrahedrally coordinated by four methionine residues, two of them being from one monomer and the other two from the other monomer [8]. If a similar situation also applies for the *Hp*CrdA, the binding of the copper ion could involve some of the conserved methionine residues and additional ones (not present in other homologs) that form a nice sulfur cluster close to the exposed surface (Met69, Met72, Met75, Met78, and Met 99) (Figure 7c), which corresponds to the area where *Ps*CopC dimerizes, forming the double copper-binding site. The usual coordination for Cu^+^ (d^10^) is tetrahedral, but it can also be linear (for the coordination number, CN, of 2) and planar (CN = 3). Cu^2+^ (d^9^) can accommodate more ligands: CN = 4, square planar or distorted tetrahedral, CN = 5, square pyramidal or trigonal bipyramidal, or the most common CN = 6, octahedral, with a distortion caused by the Jahn–Teller effect—unevenly filled e_g_ or t_2g_ shells. Usually, the unpaired electron is in the d_x2-y2_ orbital, resulting in two elongated bonds and tetragonal distortion of the octahedron. In the proteins, however, the binding site often dictates more distorted coordination polyhedra and lower coordination numbers. In the described CopC structure (Figure 7a), a CN of three was found for Cu^2+^, but it is also appropriate for Cu^+^, whereas the CN of four (Figure 7b) is adequate for both oxidation states of copper. In *Hp*CrdA, the methionine-rich site is in a flexible loop and can adopt different geometries for the two copper oxidation states. It could bind copper in both oxidation states (I and II), but with different binding affinities, a micromolar was found for Cu(II) and was lower than the nanomolar proposed for Cu(I).

*Hp*CrdA could selectively bind the excess of free copper ions in both forms, Cu(I) and Cu(II), when the concentration of free copper ions in the *H. pylori* cytoplasm reaches high values. Furthermore, *Hp*CrdA is supposed to interact with other *H. pylori* copper efflux pump proteins (CrdB, HP1328, and HP1329) that transport copper outside the cell, limiting the concentration of free copper ions in the cell under toxic levels [2].

## Data Availability

Not applicable.

## Supplementary Materials

The following supporting information can be downloaded at: https://www.mdpi.com/article/10.3390/molecules27113387/s1. Figure S1. SDS-PAGE of cleaved *Hp*GSTCrdA purified by SEC with lanes 1–4 and 5–6 corresponding to the GST (molecular mass of the monomer ~26 kDa) and CrdA (molecular mass of the monomer ~12.4 kDa) fractions, respectively; Figure S2. 15N HSQC spectrum of *Hp*CrdA protein sample recorded on a 800MHz VNMRS NMR spectrometer using a HCN cryogenic probehead with inverse detection at 298K; Figure S3. Sizing of *Hp*CrdA using post-separation labelling on a microfluidic chip; Figure S4. Interaction study of Cu(I):*Hp*CrdA complex by microscale thermophoresis; Figure S5. Cartoon view of copper binding site in *Pseudomonas fluorescens* CopC (PDB ID 6NFQ); Table S1. CD data of the *Hp*CrdA analysed by the secondary structure analysis software, CDNN [20].
