# Supplementary material for: Copper Binding and Oligomerization Studies of the Metal Resistance Determinant CrdA from Helicobacter pylori
Source: Molecules. 2022 May 24;27(11):3387. doi: 10.3390/molecules27113387 (PMC9182242; doi:10.3390/molecules27113387)
Supplement: Supplementary file 1 [file molecules-27-03387-s001.zip › molecules-1727474-supplementary.pdf]

Supplementary data

Copper binding and oligomerization studies of the metal resistance determinant CrdA from *Helicobacter pylori*

Ivana Kekez[1,*], **Mihovil Faletar** [1], **Mario Kekez** [1], **Laura Cendron** [2], **Maya Wright** [3], **Giuseppe Zanotti** [4] **and Dubravka Matković-Čalogović** [1,*]

[1] University of Zagreb, Faculty of Science, Department of Chemistry, Horvatovac 102a, Zagreb, 10000, Croatia

[2] Department of Biology, University of Padua, Via Ugo Bassi 58/B, Padua, 35131, Italy

[3] Fluidic Analytics Ltd., Unit A Paddocks Business Centre, Cherry Hinton Road, Cambridge CB1 8DH, United Kingdom

[4] Department of Biomedical Sciences, University of Padua, Via Ugo Bassi 58/B, Padua, 35131, Italy

* Correspondence: email: ikekez@chem.pmf.hr, Dubravka Matković-Čalogović,
+38514606345, email: dubravka@chem.pmf.hr

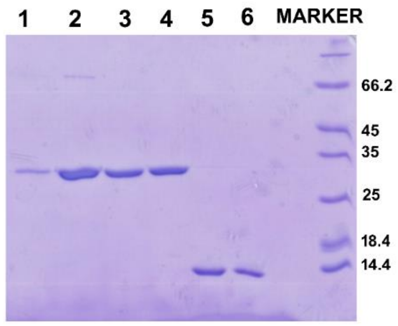

Figure S1. SDS-PAGE of cleaved *Hp*GSTCrdA purified by SEC with lanes 1 – 4 and 5 – 6 corresponding to the GST (molecular mass of the monomer ~26 kDa) and CrdA (molecular mass of the monomer ~12.4 kDa) fractions, respectively.

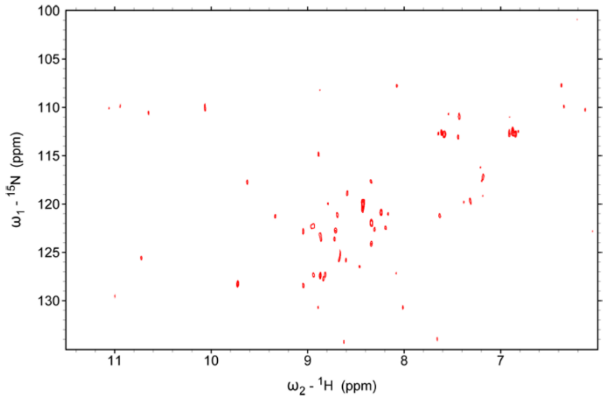

Figure S2. 15N HSQC spectrum of *Hp*CrdA protein sample recorded on a 800MHz VNMRS NMR spectrometer using a HCN cryogenic probehead with inverse detection at 298K.

Table S1. CD data of the *Hp*CrdA analysed by the secondary structure analysis software, CDNN [20]. Deconvoluted results are shown as contributions of the various components to the protein secondary structure.

SECONDARY STRUCTURE ELEMENT	%
Helix	9.0
Antiparallel β sheet	35.6
Parallel β sheet	5.2
β turn	19.5
Random coil	30.7

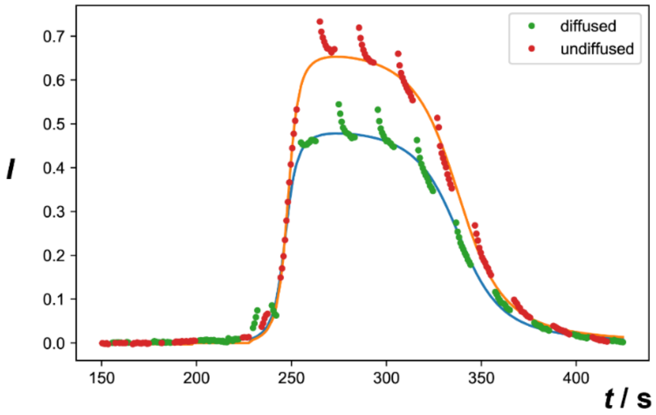

Figure S3. Sizing of *Hp*CrdA using post-separation labelling on a microfluidic chip. The y-axis shows fluorescence intensity, and x-axis time in seconds.

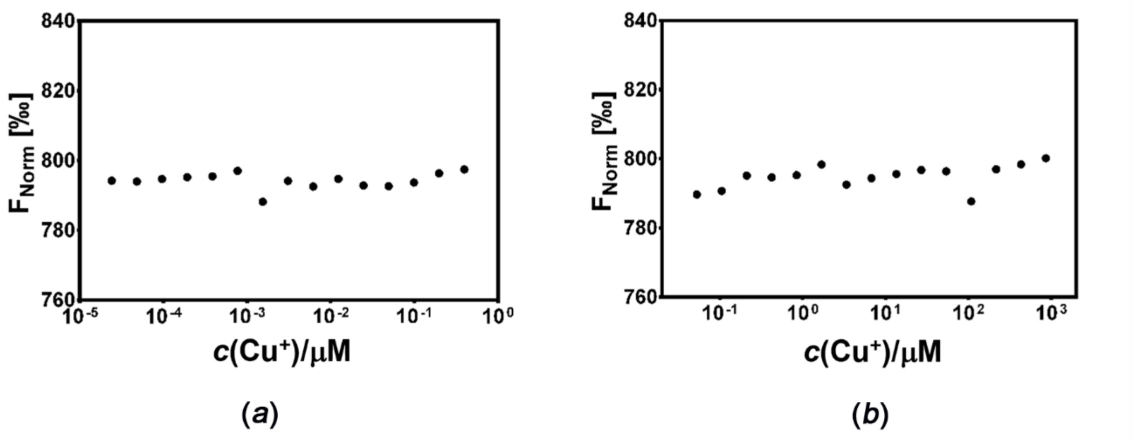

Figure S4. Interaction study of Cu(I):*Hp*CrdA complex by microscale thermophoresis; (a) lower and (b) higher copper(I) concentration range. The signal is in parts per thousand. The measurements were performed using the same protocol as for the copper(II):HpCrdA titration experiments, with the difference that only that the whole procedure including transferring to the Premium capillaries was done in a nitrogen purged glove box. Oxygen was removed from the solutions by bubbling nitrogen through them for about 10 min. A deoxygenated stock solution of 0.5 mM CuCl in 10 mM HCl and 150 mM NaCl was stored in an anaerobic glove box. For all copper(I) binding experiments both protein and copper stock solutions were diluted with assay buffer (20 mM Mops, pH 7.5, 150 mM, NaCl, 0.05% (w/v) Tween detergent) immediately before the run. The concentration of stock copper(I) chloride solution was determined by inductively coupled plasma optical emission spectroscopy on an inductively coupled plasma optical spectrometer Prodigy High Dispersion ICP (Teledyne Leeman Labs., Hudson, NH, USA).

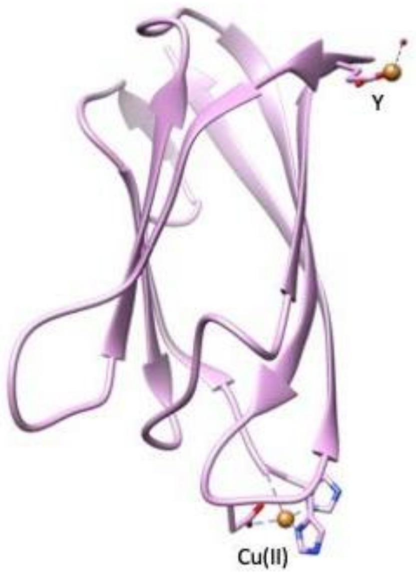

Figure S5. Cartoon view of copper binding site in *Pseudomonas fluorescens* CopC (PDB ID 6NFQ). The second position in monomer A shown in this picture is occupied by an yttrium ion (Y).
